# Flagellin Treatment Prevents Increased Susceptibility to Systemic Bacterial Infection after Injury by Inhibiting Anti-Inflammatory IL-10+ IL-12- Neutrophil Polarization

**DOI:** 10.1371/journal.pone.0085623

**Published:** 2014-01-15

**Authors:** Crystal J. Neely, Laurel B. Kartchner, April E. Mendoza, Brandon M. Linz, Jeffrey A. Frelinger, Matthew C. Wolfgang, Robert Maile, Bruce A. Cairns

**Affiliations:** 1 North Carolina Jaycee Burn Center, University of North Carolina, Department of Microbiology and Immunology, Chapel Hill, North Carolina, United States of America; 2 North Carolina Jaycee Burn Center, University of North Carolina, Department of Surgery, Chapel Hill, North Carolina, United States of America; 3 University of Arizona, Department of Immunobiology, Tucson, Arizona, United States of America; 4 University of North Carolina, Department of Microbiology and Immunology, Chapel Hill, North Carolina, United States of America,; Harvard Medical School, United States of America

## Abstract

Severe trauma renders patients susceptible to infection. In sepsis, defective bacterial clearance has been linked to specific deviations in the innate immune response. We hypothesized that innate immune modulations observed during sepsis also contribute to increased bacterial susceptibility after severe trauma. A well-established murine model of burn injury, used to replicate infection following trauma, showed that wound inoculation with *P. aeruginosa* quickly spreads systemically. The systemic IL-10/IL-12 axis was skewed after burn injury with infection as indicated by a significant elevation in serum IL-10 and polarization of neutrophils into an anti-inflammatory (“N2”; IL-10^+^ IL-12^−^) phenotype. Infection with an attenuated *P. aeruginosa* strain (ΔCyaB) was cleared better than the wildtype strain and was associated with an increased pro-inflammatory neutrophil (“N1”; IL-10^−^IL-12^+^) response in burn mice. This suggests that neutrophil polarization influences bacterial clearance after burn injury. Administration of a TLR5 agonist, flagellin, after burn injury restored the neutrophil response towards a N1 phenotype resulting in an increased clearance of wildtype *P. aeruginosa* after wound inoculation. This study details specific alterations in innate cell populations after burn injury that contribute to increased susceptibility to bacterial infection. In addition, for the first time, it identifies neutrophil polarization as a therapeutic target for the reversal of bacterial susceptibility after injury.

## Introduction

Each year traumatic injury accounts for over 40 million emergency room visits and 2 million hospital admissions across the United States [1]. Severe trauma predisposes patients to infection with rates as high as 37% of patients [2]. Infectious complications, such as sepsis and pneumonia, increase the length of hospitalization and cost of treatment [3], [4]. Furthermore, infection increases a traumatically injured patient's mortality rate by 5-fold [5].

It is clear that severe burn-injury results in a complex interaction of both innate and adaptive immunity that leads to immune dysfunction, infection and often sepsis. Much work has been focused on defining alterations in the adaptive immune system, with T cell apoptosis [6], [7], lymphopenia [8], T cell cytokine polarization [9]–[12] and upregulation of regulatory T cell (T_reg_) suppressive function [13]–[15] being key players. However, in healthy individuals, the innate immune system is sufficient for clearing most invading bacteriaNeutrophils, which are considered the first-responders of the innate immune system, have a wide variety of anti-microbial functions including phagocytosis, release of granule proteins, and generation of neutrophil extracellular traps (NETs) [16]–[18]. Macrophages and dendritic cells are also phagocytic, and antigen presentation and pro-inflammatory cytokine secretion (such as TNF-α and IL-12) by these cells induce and shape the adaptive immune response [19], [20]. Toll-like receptors (TLRs), which recognize conserved microbial products, are vital for detection of invading pathogens. TLR signaling leads to the induction or suppression of hundreds of inflammatory genes that further influence an immune response [21], [22]. Collectively, these innate immune responses lead to clearance of invading bacteria.

During sepsis, defective bacterial clearance has been linked to alterations in the innate immune response. TLR expression and signaling is often altered leading to hypo- or hyper-responsiveness [23], [24]. In addition, macrophages and neutrophils, which are extremely plastic, tend to be polarized into an anti-inflammatory phenotype due to TLR-signaling by danger-associated molecular patterns (DAMPS) released from damaged tissue [25]–[29]. These polarized macrophage (M2) and neutrophil (N2) cells secrete high amounts of IL-10, a potent anti-inflammatory cytokine and have been implicated in burn injury [30]–[33]. IL-10 can limit tissue damage by dampening the exaggerated production of pro-inflammatory cytokines observed during sepsis and induce tissue healing [34], [35]. However, excessive IL-10 has been shown to be detrimental for bacterial clearance by attenuating protective pro-inflammatory cytokines, such as IL-12 [36]–[38]and correlates with worse outcome after burn injury [39]. Additionally, in various models of trauma a Ly6g^+^ CD11b^+^ myeloid cell population has been shown to arise [40], [41] which are thought to be analogous to the Myeloid-derived Suppressor Cells (MDSC) that mediate immune suppression in the tumor microenvironment although their role in injury is controversial [40], [42]. We hypothesized that these innate immune modulations observed during sepsis also contribute to increased bacterial susceptibility after severe trauma.

Utilizing a well-established murine model of burn injury to replicate infection following trauma, we found that burn mice were highly susceptibility to systemic wildtype *P. aeruginosa* infection after wound inoculation. The systemic IL-10/IL-12 axis was skewed after burn injury and infection demonstrated by a substantial elevation in serum IL-10. Furthermore, a significant number of neutrophils, but not macrophages, were polarized into an IL-10^+^ IL-12^−^ N2 anti-inflammatory phenotype. To confirm if neutrophil polarization played a role in bacterial clearance after burn injury, mice were then infected with attenuated *P. aeruginosa* strain (Δ*CyaB*). We found that better clearance of Δ*CyaB* compared to the wildtype strain was associated with an increased N1 response in burn mice. Also, we were able to skew the neutrophil response towards a pro-inflammatory N1 phenotype by the administration of a TLR5 agonist, flagellin, immediately after burn injury that correlated with an increased clearance of wildtype *P. aeruginosa* after wound inoculation.

These findings, for the first time, detail specific alterations in innate cell populations after burn injury that contribute to increased susceptibility to bacterial infection and reveal neutrophil polarization as a therapeutic target for the reversal of bacterial susceptibility after injury.

## Methods

### Animals

Wildtype C57BL/6 (B6) mice were purchased from Taconic Farms (Hudson, NY). All mice used in the study were maintained under specific pathogen-free conditions in the Animal Association of Laboratory Animal Care-accredited University of North Carolina Department of Laboratory Animal Medicine Facilities. All protocols were approved by the University of North Carolina Institutional Animal Care and Use Committee and performed in accordance with the National Institutes of Health guidelines.

### Mouse Burn Injury

Eight to 12 week old (>18 grams), female B6 mice were used for all experiments. Animals were anesthetized by inhalation of vaporized isoflurane (Baxter Healthcare, Deerfield, IL) and had their dorsal and flank hair clipped. A subcutaneous injection of morphine (3 mg/kg body weight; Baxter Healthcase) was given prior to burn injury for pain control, and an intraperitoneal injection of lactated Ringer's solution (0.1 mL/g body weight; Hospira, Lake Forest, IL) was given immediately after burn injury for fluid resuscitation. To create a full-contact burn of approximately 20% total body surface area (TBSA), a 65 g rod copper rod (1.9 cm in diameter), heated to 100°C was used. Four applications of the rod, each for 10 seconds, to the animal's dorsal/flank produced the wound. Previous studies analyzing skin biopsies of the burn wound have demonstrated full-thickness cutaneous burn with visible unburned muscle beneath when following this procedure. Animals were returned to individual cages, provided food and morphinated water *ab libitum*, and monitored twice a day. Sham controls with 0% TBSA underwent all described interventions except for the actual burn injury. There was negligible mortality (<1%) after burn injury alone.

### Bacterial strains and preparation

A wildtype strain (PAK) and a mutant strain (Δ*CyaB*) of *P. aeruginosa* were obtained from M. Wolfgang (University of North Carolina, Chapel Hill, NC) [43]. Bacteria were grown from frozen stock at 37°C overnight in Luria-Bertani (LB) broth then transferred to fresh medium and grown for an additional 2 hours or until mid-log phase. The cultures were centrifuged at 13,000 rpm for 30 seconds, and the pellet washed with 1 mL of 1 % protease peptone in phosphate buffered saline (PBS +1%PP). Following a second wash, the bacterial concentration was determined by assessing optical density at 600 nm. After diluting the bacteria to obtain the desired concentration, the inoculum was verified by plating serial 10-fold dilutions on LB agar plates containing 25 ug/L irgasan (Sigma-Aldrich, St. Louis, MO).

### Animal infections

Twenty-four hours following burn or sham injury, mice were anesthetized by intraperitoneal injection of Avertin (0.475 mg/g body weight; Sigma-Aldrich). A subcutaneous injection of bacteria was injected in the mid-dorsal region. For burn mice, this was in unburned skin surrounded by burn wound. Initial experiments monitored survival until 120 hours post infection (hpi). In subsequent experiments, mice were sacrificed at 48hpi to enumerate bacterial load and analyze immune responses. In select experiments, mice were administrated flagellin two hours prior to infection. Ultrapure flagellin from *S. typhumurium* (InvivoGen; San Diego, CA) was given intraperitoneally at a concentration of 0.125 ng/100 ul per mouse.

### Determination of bacterial infection

At time of sacrifice, a 5 mm skin biopsy of the initial injection site, the left lobe of the liver, and the lungs were aseptically removed and placed in 0.5 mL of LB broth. The tissues were homogenized using 3.2 mm stainless steel beads and a BulletBlender (Next Advance; Averill Park, NY). Serial dilutions of tissue homogenates were plated on LB agar containing irgasan and incubated overnight at 37°C.

### CD11b+ cell enrichment

Cells suspensions were prepared from spleens of mice. CD11b^+^ cells were positively selected using the BD Imag Mouse CD11b Magnetic Particles according to manufacturer's instructions (BD Biosciences). This method routinely provided a highly enriched population of CD11b^+^ cells.

### In vitro stimulation

Following the CD11b enrichment, both CD11b^+^ and CD11b^−^ cells were resuspended in RMPI containing 10% fetal bovine serum to achieve 10^6^cells/mL. Cells were plated in a 48 well plate and cultured for 6 hours with 0.1 ng/mL of LPS (Sigma-Aldrich) at 37°C at 5% CO_2_. During the last 4 hours of the stimulation, 3.0 ul/mL of brefeldin-A solution (eBioscience; San Diego, CA) was added to block protein secretion.

### Flow cytometric analysis

Splenocytes were incubated with anti-mouse CD16/32 (BD Biosciences; San Jose, CA) at a concentration of 1 ug per million cells for 5 min at 4°C to block Fc receptors. The panel of mAbs used for flow cytometric analyses were anti-Gr1 (RD-8C5), anti-CD11b (M1/70), anti-Ly6C (AL21), anti-Ly6G (1A8), anti-CD11c (N418), anti-F4/80 (BM8), anti-NK1.1 (PK136), anti-TLR2 (6C2), anti-TLR4 (MTS510), anti-TLR5 (85B152.5), anti-IL-10 (JES5-E16E3), and anti-IL-12 (p40/p70), which were purchased from BD Biosciences, eBiosciences, and BioLegend (San Diego, CA). Intracellular staining was performed using BD Cytofix/Cytoperm Kit (BD Bioscience). Data were collected on a Dako CyAN and analyzed using Summit software (Dako; Carpinteria, CA).

### Serum cytokine analysis

Submandibular bleeds were performed on mice prior to organ harvest. Serum was collected using MicroTubes with gel and following manufacturer's protocol (IRIS International, Westwood, MA). Serum IL-10 and IL-12 levels were determined using the BD Cytometric Bead Array (Becton Dickinson, San Diego, CA).

### Statistical analysis

Data were analyzed using Student's *t* test for differences in CFU recovery, cell staining, and cytokine assays; log-rank analysis was used to test differences in mouse survival; two way ANOVA with Tukey post-test was used to test differences in TLR expression GraphPad Prism version 5 was used for the analyses. Statistical significance was defined as *p*≤0.05 unless indicated otherwise.

## Results

### Burn mice, but not sham mice, developed a systemic infection following wound inoculation with wildtype *P. aeruginosa*

**Figure 1:**
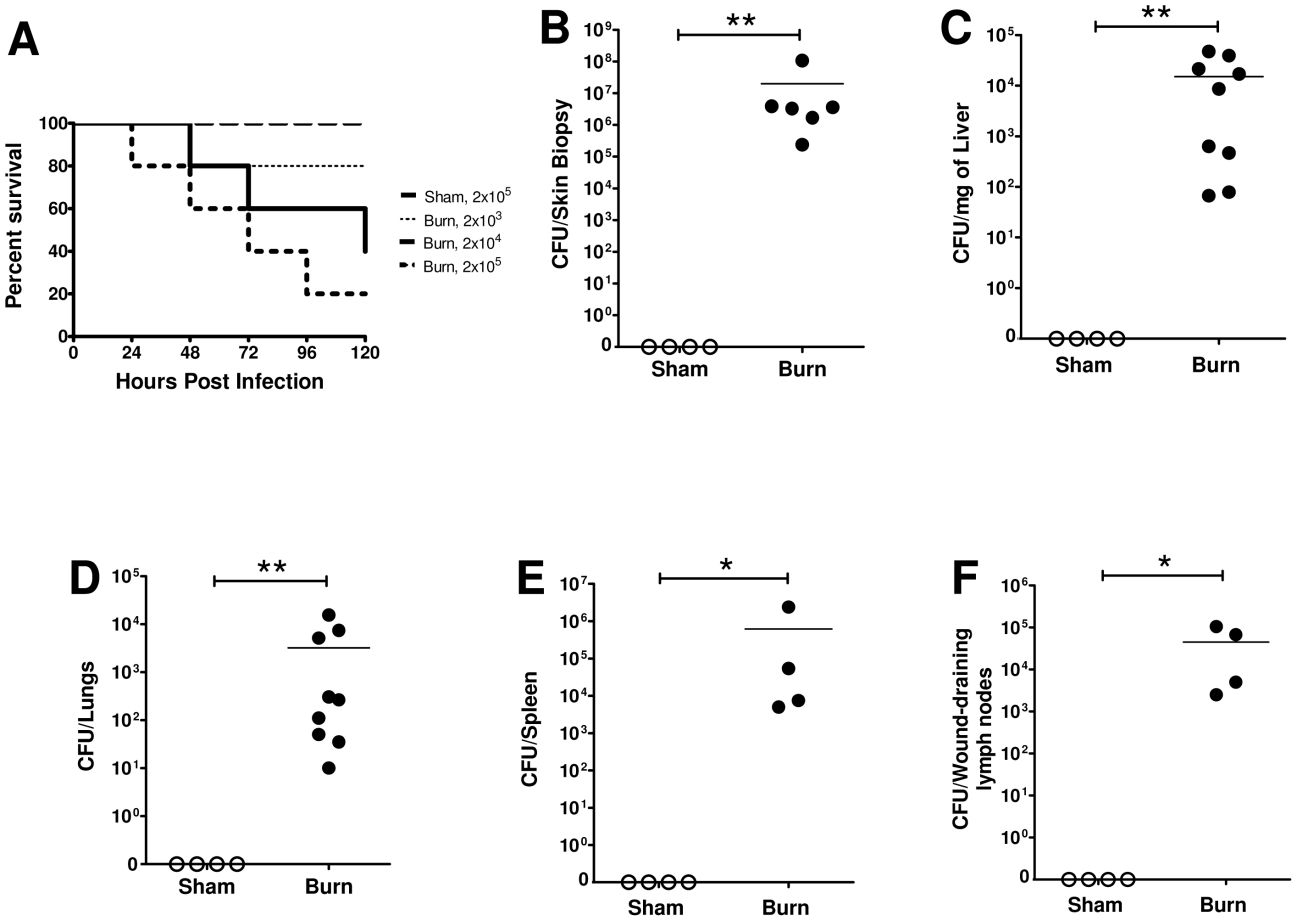
Burn mice, but not sham mice, exhibit dose-dependent mortality and develop a systemic infection following a *P. aeruginosa* wound inoculation. Wildtype *P. aeruginosa* (PAK) was administered subcutaneously at 24 hours after burn or sham treatment. A) Various doses of bacteria (2×10^3^, 2×10^4^, or 2×10^5^ CFU/100 ul) were given and survival was monitored for 120 hours post infection (hpi). B–F) Using a dose of 2×10^4^ CFU, bacterial load at the injection site and distal organs was assessed at 48 hpi in sham (open circles) and burn (closed circles) mice. (n = 4–9 per group) *, p≤0.05. **, p≤0.005. These experiments were repeated three times with similar results.

Initial studies assessed survival of burn and sham mice following wound infection with a wildtype strain of *P. aeruginosa*, PAK. At 24 hours following burn or sham procedure, mice were anesthetized and given a subcutaneous injection of bacteria (2×10^3^, 2×10^4^, or 2×10^5^ CFU/100 ul) at the mid-dorsum. There was 100% survival of sham mice, even with the highest dose of 2×10^5^ CFU (Figure 1A). Burn mice, however, exhibited mortality that was dose dependent (Figure 1A). Mortality of infected burn mice began as early as 1 day after inoculation. To evaluate bacterial clearance in burn and sham mice various tissues were harvested 48 hours following infection (2×10^4^ CFU/100ul), which was before significant mortality occurred. As shown in Figure 1B, sham mice had no bacteria recovered from skin biopsies of the injection site while all burn mice had bacteria detected. Furthermore, the amount of bacteria recovered from the skin of burn mice was 1–4 logs higher than the initial inoculum. This suggests bacterial recovery was not solely due to a lack of clearance, but that bacteria were actively replicating in the skin. Distal organs were also analyzed to examine bacterial dissemination. The liver, lungs, wound-draining lymph nodes and spleen of sham mice had no detectable bacteria, whereas the organs of burn mice had a high bacterial load (Figure 1C–1F). These data show that burn mice develop a systemic infection by 48 hours following wound inoculation with a wildtype strain of *P. aeruginosa* (PAK).

### Innate cell populations had altered TLR expression with the combination of burn injury and infection

**Figure 2:**
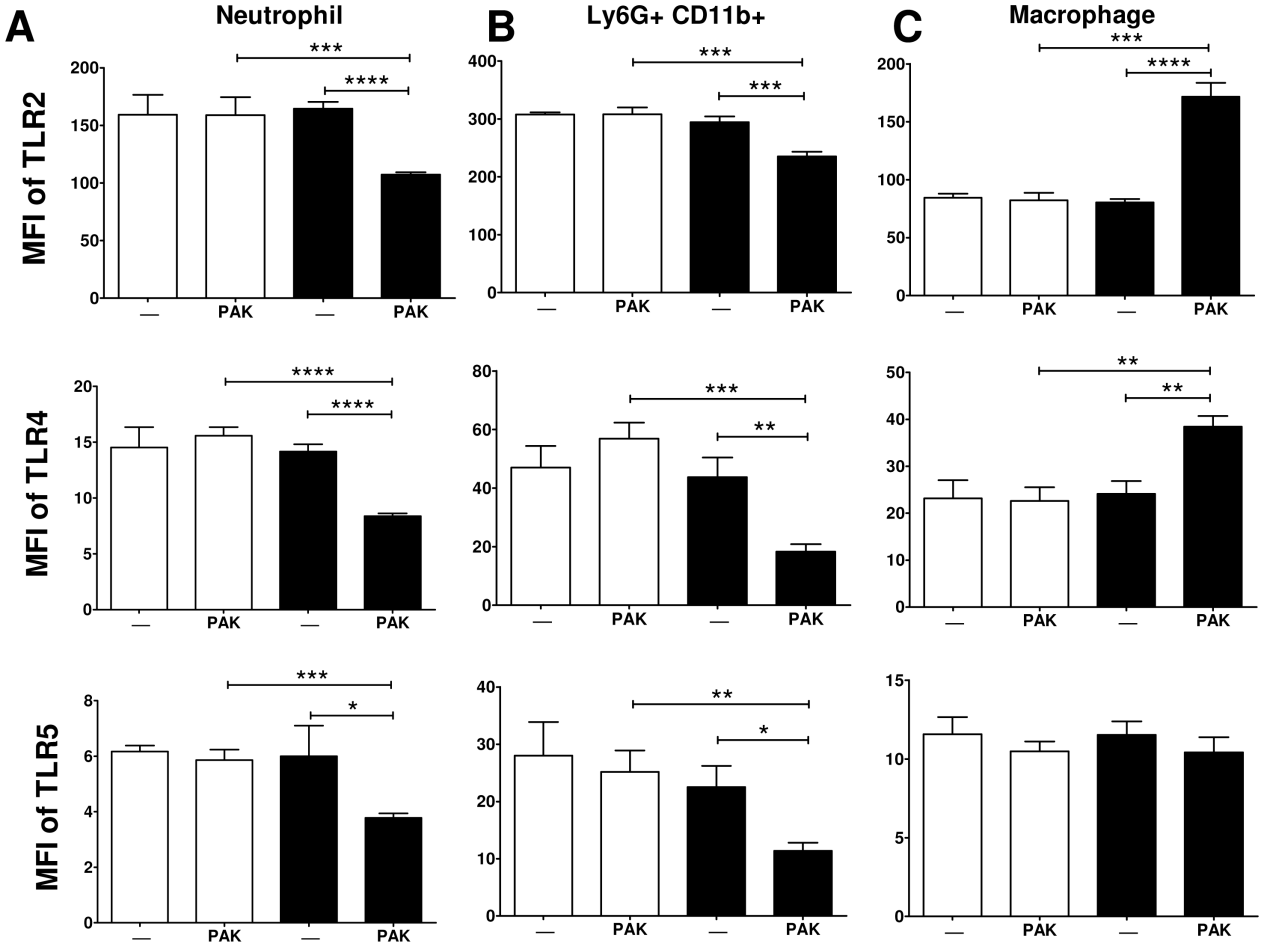
TLR expression is decreased on splenic neutrophils and Ly6G^+^ CD11b^+^ myeloid cells, but not macrophages, after burn injury with infection. Splenocytes were harvested and mean fluorescence intensity (MFI) of TLR2, TLR4, and TLR5 expression was elevated on splenic A) neutrophils, b) Ly6G^+^ CD11b^+^ cells, and B) macrophages at 3 days post burn (solid) or sham (open) treatment combined with (PAK) or without (-) *P. aeruginosa* wound inoculation. Data expressed as mean ± SEM. (n = 4–10) *, p≤0.05. **, p≤0.005. ***, p≤0.0005. ****, p<0.0001 by two way ANOVA with Tukey post test. These experiments were repeated three times with similar results.

We and others have shown that Toll-like receptor (TLR) mRNA[30], [44] and protein [30] levels changes after burn injury. Since TLR2, TLR4, and TLR5 are involved in control of *P. aeruginosa* infection by recognizing outer membrane lipoproteins, LPS, and flagellin, respectively [45]–[47], we hypothesized that decreased bacterial clearance after burn injury was due to reduced expression of these TLRs on innate immune cells. In order to perform a systemic and detailed quantification of various immune cell populations after burn injury and infection, we devised a flow cytometric staining panel to differentiate between innate cell populations (Neutrophils, Gr1+, Ly6C+, Ly6G+, CD11b+, CD11c+, F4/80-; macrophages, Gr1+, Ly6C+, Ly6G-, CD11b+, CD11c-, F4/80+; dendritic cells, Gr1-, Ly6C-, Ly6G-, CD11b+, CD11c+, F4/80- and Gr1+ myeloid MDSC, Gr1+, Ly6C+, Ly6G+, CD11b+, CD11c-, F4/80-). The absolute number of these innate populations were similar in all treatment groups (data not shown). Adaptive cell populations (T and B cell) were also largely unchanged (data not shown), with a significant decrease in CD8 T cell number only upon injury, as we have documented before [8]. Upon bacterial infection, splenic neutrophils and Ly6G^+^ CD11b^+^ myeloid cells from burn mice had significantly reduced TLR2, TLR4 and TLR5 expression compared to uninfected burn and infected sham mice (Figure 2A and 2B). In contrast, splenic macrophages from infected burn mice had increased TLR2 and TLR4 but unchanged TLR5 expression compared to uninfected burn and infected sham mice (Figure 2C). These data demonstrate that on specific innate cell populations there are acute alterations in TLR expression in response to bacterial infection after burn injury.

### Infection following burn injury resulted in a systemic increase in IL-10

**Figure 3:**
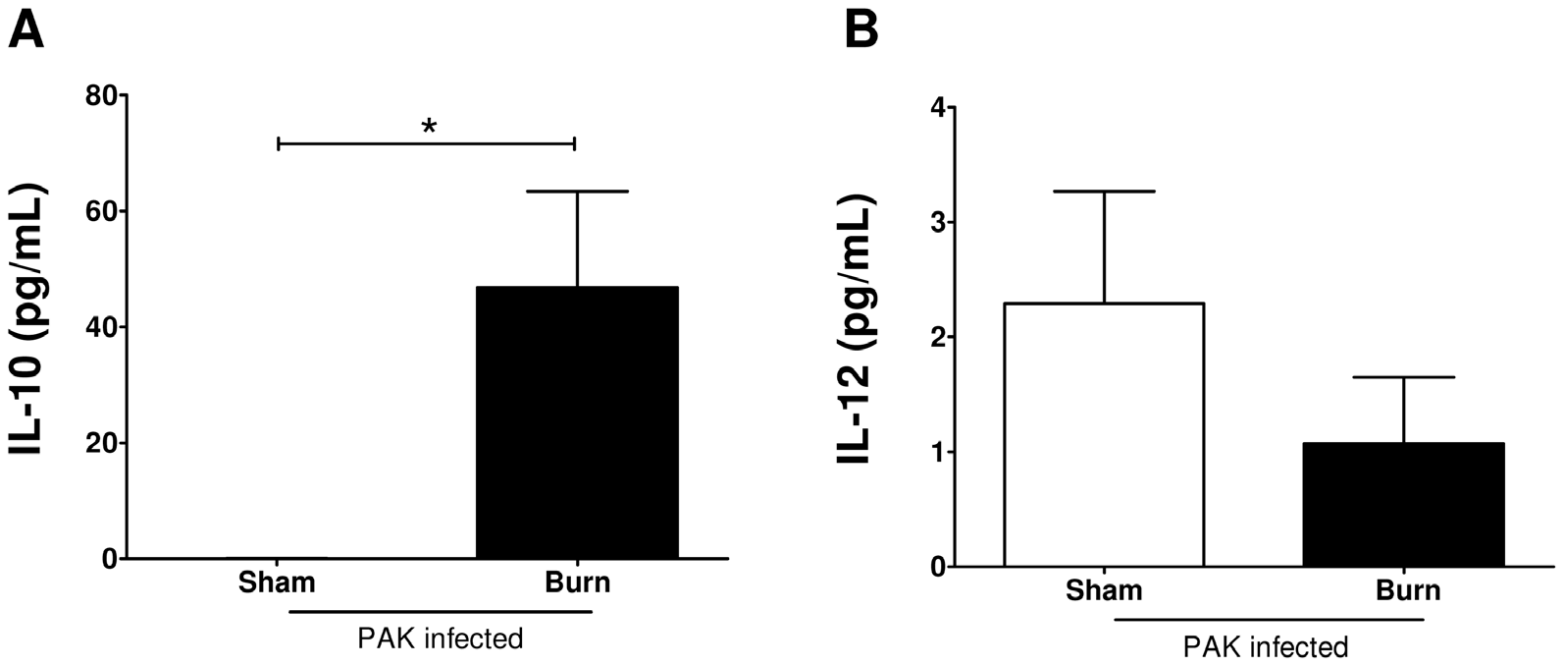
Burn mice, but not sham mice, mount a robust serum IL-10 response after *P. aeruginosa* wound inoculation. Twenty-four hours after sham (open) or burn (solid) treatment, mice were given a subcutaneous injection of wild-type *P.aeruginosa* PAK. Forty-hours following infection, serum was collected to determine circulating levels of A) IL-10 and B) IL-12 by cytometric bead array. Data expressed as mean ± SEM. (n = 10–15) *, p≤0.05.

Many studies have shown that IL-10 is deleterious whereas IL-12 is beneficial for clearance of *P. aeruginosa* [36]–[38]. Therefore, we hypothesized that infected burn mice would have a skewing in the IL-10/IL-12 axis towards an IL-10 response. Three days following burn or sham treatment, there was no detectable IL-10 in the serum (data not shown). Infection of burn mice resulted in a substantial elevation of serum IL-10 while infection of sham mice did not induce an IL-10 response (Figure 3A). Burn and sham mice also had no detectable IL-12 at three days post treatment (data not shown). However, infection caused an increase in serum IL-12 for both groups of mice (Figure 3B). In summary, infection following burn injury led to a predominant systemic IL-10 response, while infection after sham treatment induced an IL-12 response.

### Infected burn mice had an increased polarization of neutrophils, but not macrophages, into an IL-10^+^ IL-12^−^ phenotype

**Figure 4:**
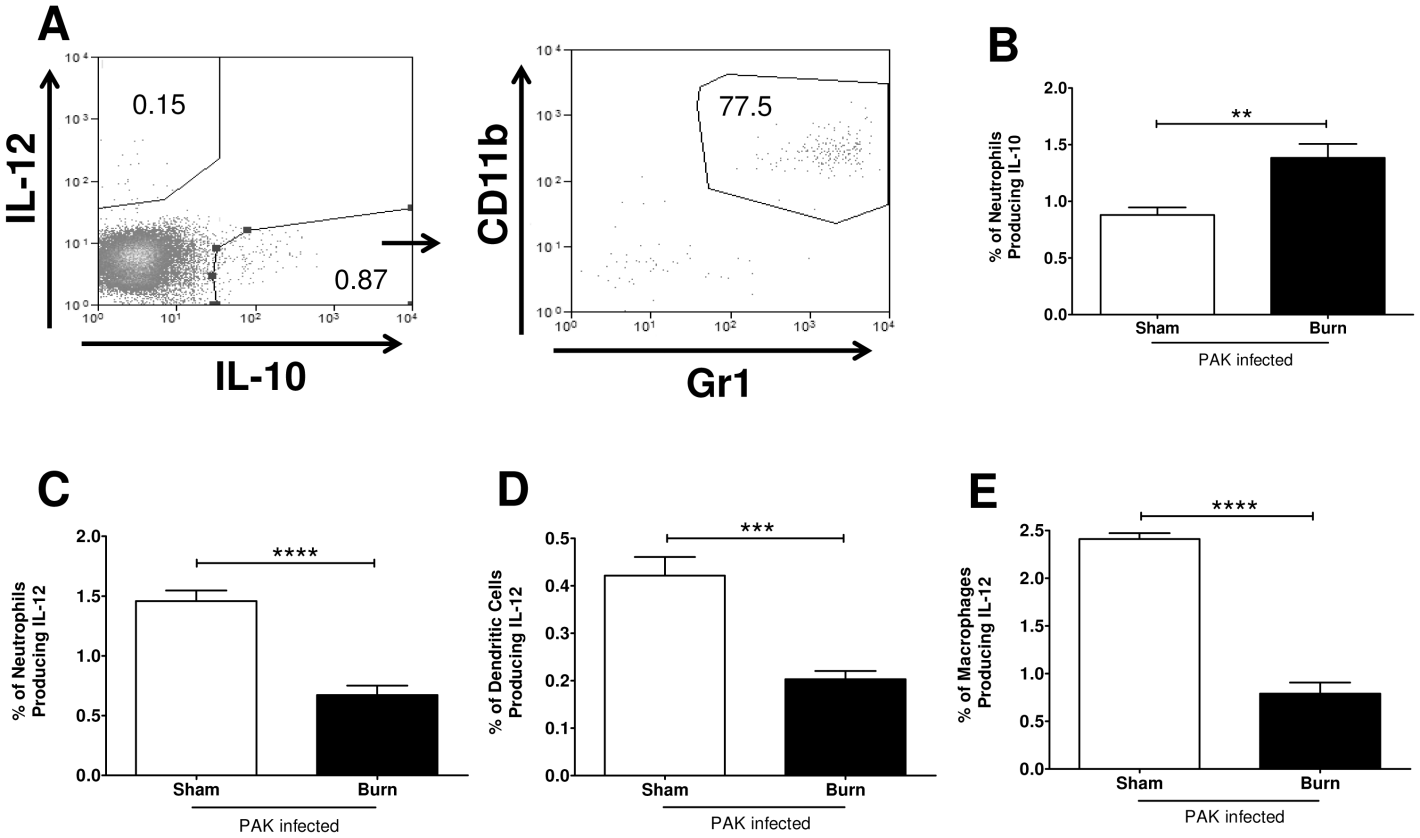
Infected burn mice have a higher percentage of IL-10^+^ neutrophils and a lower percentage of IL-12^+^ neutrophils, dendritic cells, and macrophages than infected sham mice. A) Splenocytes were harvested at 48 hours post infection and underwent intracellular staining for cytokine analysis without further stimulation *in vitro*. Shown is a representative histogram from an infected burn mouse, which indicates that IL-10 is being produced by Gr1^+^ CD11b^+^ cells within the spleen. B–E) Splenocytes were collected at 48 following infection and underwent CD11b enrichment by magnetic selection. CD11b^+^ cells were cultured in the presence of LPS and brefeldin-A then were subjected to cell surface and intracellular staining. Percentage of B) IL-10^+^ neutrophils, as well as IL-12^+^ C) neutrophils, D) dendritic cells, and E) macrophages were measured for infected sham (open) and burn (solid) mice. Data expressed as mean ± SEM. (n = 6, 7) **, p≤0.005. ***, p≤0.0005. ****, p<0.0001. These experiments were repeated three times with similar results.

Macrophages and neutrophils can be polarized into pro- (M1/N1) and anti-inflammatory states (M2/N2) [25]–[28] after TLR stimulation, particularly in the context of injury where there is release of tissue DAMPs [29]. Infected burn mice had a systemic anti-inflammatory response following infection, which was marked by elevated serum IL-10 levels; therefore, we hypothesized the innate cells were polarized towards an anti-inflammatory phenotype (IL-10^+^ IL-12^−^) following burn and infection. Splenocytes were harvested at 48 hours post infection and underwent intracellular staining for cytokine analysis without further stimulation *in vitro*. IL-10 producing Gr1+ CD11b^+^ cells were readily detected in the spleen of the infected burn mice (representative histogram, Figure 4A). Due to these data along with previous reports about IL-10 production by innate cells following burn injury [32], [48], we focused our subsequent studies on these cell types. Splenocytes were harvested at 48 following infection then underwent CD11b enrichment by magnetic selection. CD11b^+^ cells were cultured in the presence of LPS and brefeldin-A to measure intracellular accumulation of IL-10 and IL-12. Cell surface, intracellular staining and side/forward scatter indicated that neutrophils (Gr1+, Ly6C+, Ly6G+, CD11b+, CD11c+, F4/80-),but not macrophages (Gr1+, Ly6C+, Ly6G-, CD11b+, CD11c-, F4/80+), were the major immune cell type producing IL-10 in the spleen. Furthermore, infected burn mice had a significantly higher percentage of splenic neutrophils producing IL-10 than infected sham mice (Figure 4B). As for IL-12 production, infected burn mice had a significantly lower percentage of splenic neutrophils, dendritic cells, and macrophages producing this cytokine than infected sham mice (Figure 4C–E). These data, along with the serum cytokine response, suggest that following burn injury, the immune system mounts an inappropriate systemic IL-10 response with neutrophils exhibiting a N2 phenotype upon bacterial infection.

### Increased resistance of burn mice to systemic infection with an attenuated strain (ΔCyaB) *P. aeruginosa* correlated with reduced N2 polarization of neutrophils

**Figure 5:**
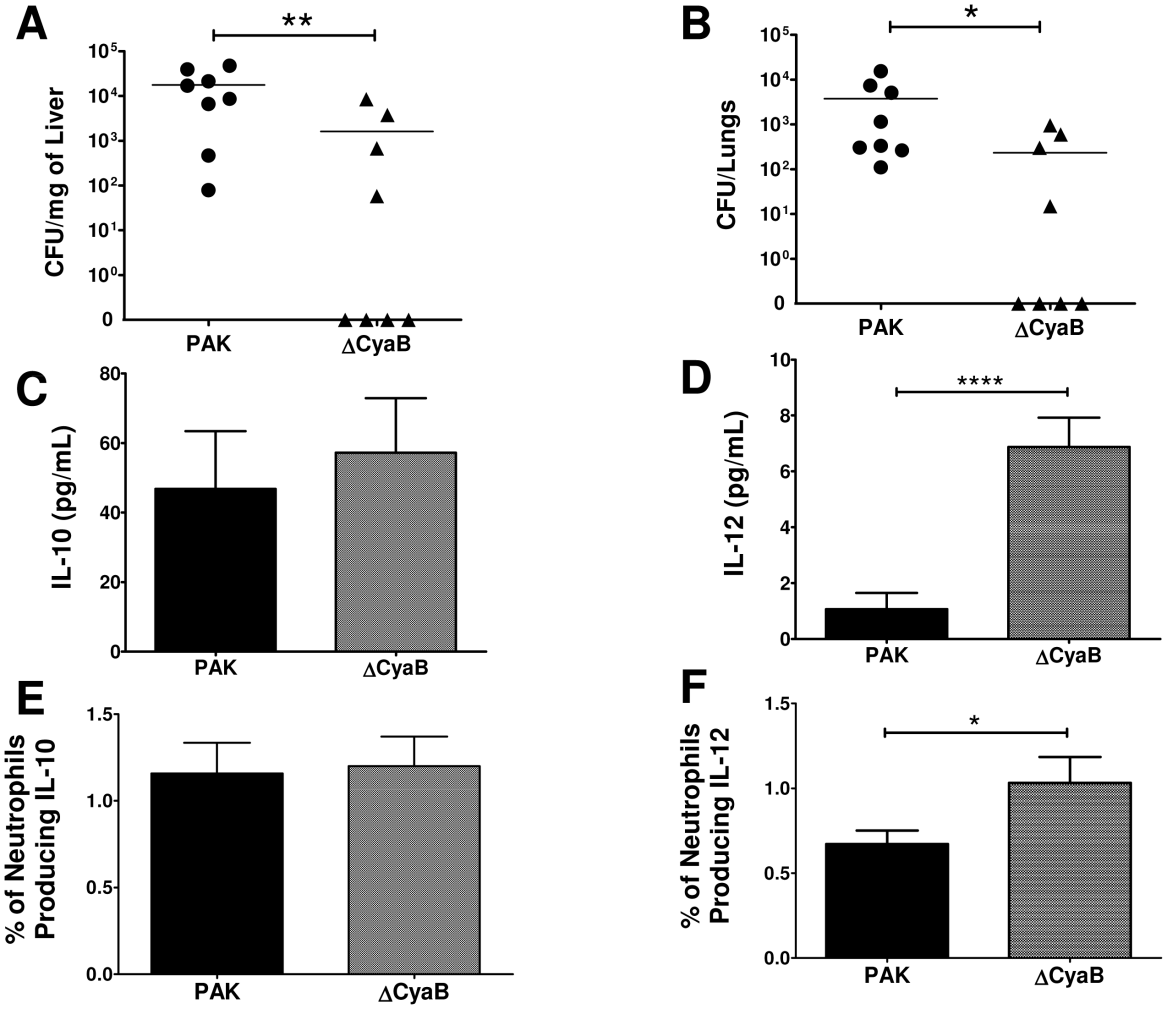
Reduced bacterial load at distal organs following wound inoculation with an attenuated *P. aeruginosa* strain (Δ*CyaB*) is associated with an increased serum IL-12 and pro-inflammatory neutrophil (N1; IL-10^−^IL-12^+^) response in burn mice. Forty eight hours following wildtype PAK (circles/solid bars) or Δ*CyaB* (triangles/checkered bars) wound infection, various organs were harvested from burn mice. Bacterial load in A) liver and B) lung samples was determined by colony forming unit (CFU) assay. Serum C) IL-10 and D) IL-12 levels were assessed by cytometric bead array. Also, the percentage of splenic neutrophils producing E) IL-10 and F) IL-12 was determined by flow cytometric analysis. Data expressed as mean ± SEM. (n = 8, 8) *, p≤0.05. **, p≤0.005. ****, p<0.0001. These experiments were repeated three times with similar results.

Δ*CyaB* is a mutant strain of *P. aeruginosa* that has been previously reported to be attenuated in an adult mouse model of acute pneumonia [49]. We predicted that burn mice could control infection with Δ*CyaB* better than wildtype PAK. Also, we hypothesized that any differences in the innate immune response between Δ*CyaB* and PAK infection would reveal mechanisms that contribute to enhanced bacterial clearance and thus identify potential targets for immune modulation. Twenty-four hours following burn or sham treatment, mice were given a subcutaneous injection of wildtype PAK or Δ*CyaB* mutant (2×10^4^ CFU/100 ul). At 48 hours following infection, skin biopsies at the injection site were harvested to measure localized bacterial clearance. Distal organs were also harvested to assess bacterial dissemination from the injection site. Regardless of *P. aeruginosa* strain, infected sham mice had no bacteria recovered from their skin, liver, and lung samples (data not shown). Infection with PAK or Δ*CyaB* following burn injury resulted in a similar bacterial load at the injection site (data not shown). In contrast, burn mice infected with Δ*CyaB* had significantly less bacterial recovery in the distal organs than burn mice infected with PAK (Figure 5A and 5B). These data indicate that burn mice are more resistant to developing systemic infection with an attenuated strain of *P. aeruginosa* than with wildtype PAK. Therefore, burn mice retain some antibacterial activity which allows for improved control of the attenuated strain.

Infection of burn mice, regardless of bacterial strain, caused an elevation of serum IL-10 compared to sham mice (Figure 5C). In contrast to PAK, infection with Δ*CyaB* following burn injury resulted in a significant increase in serum IL-12 levels (Figure 5D). In both treatment groups, the main source of IL-10 in the spleen was neutrophils. Also, the percentage of splenic neutrophils producing IL-10 was similar in burn mice infected with Δ*CyaB* and those infected with PAK (Figure 5E). Δ*CyaB* infection also resulted in a higher percentage of IL-12^+^ neutrophils within the spleen (Figure 5F). Hence, infection with Δ*CyaB* following burn injury results in a higher percentage of IL-12^+^ cells within the spleen and an increase in serum IL-12. TLR2, TLR4, and TLR5 expression on the various immune cells was comparable between PAK and Δ*CyaB* infected burn mice (data not shown). These data suggest that the reduced susceptibility to Δ*CyaB* in the burn mice is due to a skewing of the IL-10/IL-12 balance to a protective IL-12 response.

### Treatment of mice with flagellin after burn injury enhanced clearance of wildtype *P. aeruginosa*

**Figure 6:**
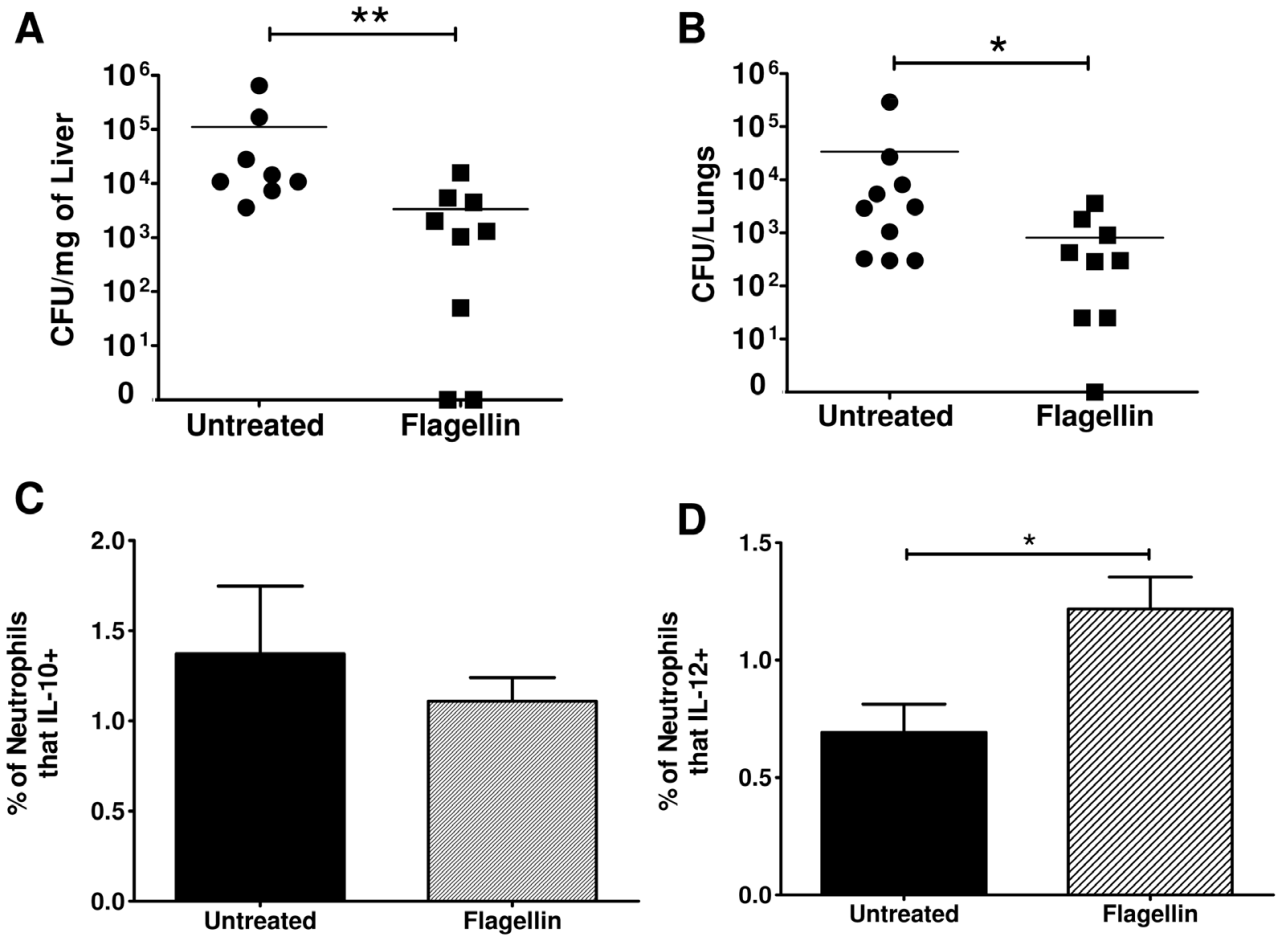
Administration of flagellin at burn resuscitation and prior to wound infection with wildtype *P. aeruginosa* (PAK) reduces bacterial load in the periphery and increases the percentage of IL-12 producing neutrophils within the spleen. Burn mice were given given an intraperitoneal injection of flagellin (circles/solid bars) or left untreated (squared/striped bars) twenty-two hours after burn injury. Twenty-four hours after burn injury, mice were challenged with subcutaneous wound infection with PAK. Forty-eight hours following the bacterial challenge, various organs were harvested. Bacterial load in A) liver and B) lung samples was determined by colony forming unit (CFU) assay. The percentage of splenic neutrophils producing C) IL-10 and D) IL-12 was determined by flow cytometric analysis. Data expressed as mean ± SEM. (n = 8–10) *, p≤0.05. **, p≤0.005. These experiments were repeated three times with similar results.

Flagellin, the ligand of TLR5, has been shown to increase IL-12 production [29], [50]. Therefore, we hypothesized that flagellin could improve clearance of wildtype *P. aeruginosa* (PAK) in burn mice by increasing the protective IL-12 response. Burn mice were resuscitated after burn injury and received an intraperitoneal injection of flagellin (0.125 ng/100 ul) twenty-hours later. Twenty-four hours after burn they were then infected subcutaneously with PAK. Forty-eight hours following infection with or without treatment with flagellin, various organs were harvested to determine bacterial load. Pretreatment with flagellin did not affect bacterial recovery from skin biopsies at the injection site (data not shown). However, there were significantly less bacteria recovered from the liver and lungs of burn mice pretreated with flagellin compared to untreated controls (Figure 6A and 6B). The reduced bacterial load in the periphery correlated with an increased percentage of IL-12 producing neutrophils whereas IL-10 production by neutrophils was unchanged (Figure 6C and 6D). The absolute number of innate and adaptive cells were unchanged between flagellin and flagellin-untreated burn mice. These data suggest that a single treatment with flagellin after injury is sufficient to reduce the systemic infection of wildtype *P. aeruginosa* by skewing more neutrophils towards a pro-inflammatory phenotype.

## Discussion

Severe trauma results in a period of immune impairment that predisposes the patient to infectious complications, such as sepsis. However, the specific mechanisms that contribute to diminished bacterial clearance are not clearly defined. In this study, we utilized a murine model of severe burn injury and challenged mice with a clinically relevant pathogen to reveal specific trauma-induced deviations in the innate immune response that contribute to increased susceptibility to infection. Within 48 hours of wound inoculation with a wildtype strain of *P. aeruginosa* (PAK), bacteria replicate to a high titer and spread to distal organs resulting in bacterial sepsis. Neutrophils and Ly6g^+^ CD11b^+^ myeloid cells have decreased TLR expression. In addition, the neutrophils are profoundly polarized into an anti-inflammatory (“N2”; IL-10^+^ IL-12^−^) phenotype.

Furthermore, we hypothesized that some antimicrobial effector functions are retained after severe burn injury and that amplifying these responses therapeutically can enhance bacterial clearance even if in face of overt immune suppression. To identify these potential targets, mice were infected with an attenuated strain of *P. aeruginosa* (Δ*CyaB*). We found that burn mice have greater control of ΔCyaB infection than wildtype PAK infection, which is exhibited by reduced bacterial recovery systemically. By comparing various aspects of the innate immune response, it appears that increased neutrophil polarization towards a pro-inflammatory phenotype (N1; IL-12^+^ IL-10^−^) is responsible for improved clearance of Δ*CyaB* in the periphery. We next investigated the effectiveness of flagellin, a natural TLR5 ligand that can induce IL-12 production, as a therapeutic agent in our model [29], [50]. We found that treatment with flagellin after burn injury enhances clearance of wildtype *P. aeruginosa* (PAK) in the periphery and increases the percentage of IL-12 producing neutrophils in the spleen. Nevertheless, IL-10 production by splenic neutrophil remained elevated compared to sham controls. These data suggest that although infection following burn injury polarizes neutrophils towards an anti-inflammatory phenotype, flagellin administration can tilt this back towards a pro-inflammatory response that is beneficial for bacterial clearance.

Previous studies have attempted to delineate cellular mechanisms underlying the increased susceptibility to infection after injury, which is a very pressing clinical need. This study utilized a very precise panel of antibodies for the flow cytometric identification of specific innate cell populations so that their role in infection after burn injury could be better assessed. Using cell surface markers CD11b, CD11c, F4/80, Gr1, Ly6C, and Ly6G, we can clearly define neutrophils (F4/80^−^ Gr1^+^ (Ly6C^+^ Ly6G^+^) CD11b^+^ CD11c^+^), macrophages (F4/80^+^ Gr1^+^ (Ly6C^+^ Ly6G^−^) CD11b^+^ CD11c^−^), a Ly6g^+^ CD11b^+^ myeloid population (F4/80^+^ Gr1^+^ (Ly6C^+^ Ly6G^+^) CD11b^+^ CD11c^−^) and dendritic cells (F4/80^−^ CD11b^+^ Gr1^−^ CD11c^+^). Using such an in depth staining panel and gating scheme allowed for quantification of various immune innate cell populations after injury that has not been reported to date.

Controversy exists as to whether the Ly6g^+^ CD11b^+^ cells that arise after trauma are analogous to the Myeloid-derived Suppressor Cells (MDSC) that mediate T cell suppression in the tumor microenvironment [40]. Our laboratory has recently described that burn-induced Ly6g^+^ CD11b^+^ cells suppress T cell proliferation [51] and polarize T cells towards a Th2-anti-inflammatory response [52] suggesting they mimic aspects of MDSC function. Ly6g^+^ CD11b^+^ cells employ various mechanisms, such as arginase, IL-10, and nitric oxide production, to inhibit T cell proliferation and activation [53]–[55]. This study did not reveal IL-10 secretion by Ly6G^+^ CD11b^+^ myeloid cells after burn injury and/or an acute bacterial infection, but we predict that these cells become a predominant population at later time points after burn injury [51] due to continuous myelopoeisis. IL-10 itself has many effects on other immune cells, including upregulation of T_reg_ suppression, decreased effector T cell function. IL-10 can also downregulate innate cell function including inhibition of reactive oxygen species vital for killing of bacteria. The innate system is thought to drive the resultant adaptive response. Further work is required to determine if and how these cells impact both the innate and adaptive arms of the immune system later after burn injury.

As for the other innate cells populations, we observed neutrophil, but not macrophage, polarization in our model system. Polarization of adaptive immune cells, such as naïve CD4^+^ T cells into a Th1 or Th2 phenotype, is well established [56]. However, the polarization and plasticity of innate immune cell populations has only been recently recognized. Most of the information within the field originates from tumor research and mainly focuses on macrophage polarization [57]. Although the details are still unclear, the literature suggests that the local microenvironment in which a cell is activated determines the cell's subsequent phenotype and that changing this microenvironment can skew polarization of the cell population. For example, a macrophage can be polarized towards a pro-inflammatory phenotype (M1) marked by production of IL-12, as well as other pro-inflammatory mediators, when activated in the presences of interferon-gamma [58]. However, if a macrophage is then exposed to IL-10, glucocorticoids, or immune complexes in the presence of the TLR ligands, it can exhibit an anti-inflammatory phenotype (M2, IL-10^+^ IL-12^−^) [59], [60]. In our model system, we find that neutrophils, not macrophages, are the main innate immune cell population that is polarized. It appears that infection following burn injury skews neutrophils towards an anti-inflammatory phenotype. Yet when mice are administered flagellin, they exhibit a mixed N1/N2 phenotype that correlates with enhanced bacterial clearance in the periphery. In the context of sepsis, a predominant M1 response is detrimental to local tissue since the robust pro-inflammatory cytokine production by the macrophages can exacerbate tissue damage [26]. Also, an overt M2 response is believed to deleterious by significantly impairing bacterial clearance [61]. Thus, a mixed M1/M2 response appears to be ideal during sepsis. Our data support the idea that a mixed N1/N2 response is also beneficial after sepsis; however, future research is needed to delineate this correlation in more detail.

Since infectious complications are a main cause of mortality after traumatic injury, it is essential to identify biomarkers of infection and drug targets to improve control of invading pathogens. Numerous studies have linked high circulating levels of IL-10 with poor outcome following burn injury, sepsis, and a wide variety of bacterial infections [36], [38], [62]. In our model system, serum IL-10 is elevated in infected burn mice, regardless of strain, but not in uninfected controls. Collectively, this supports the use of IL-10 as a useful biomarker of bacterial infection.

Taken together these data detail specific changes in innate cell populations following burn injury that contribute to increased susceptibility to bacterial infection and reveal neutrophil polarization as a therapeutic target for the reversal of bacterial susceptibility after injury. Future experiments should examine other aspects of neutrophil function, such as phagocytosis and NET formation, after burn injury and infection and determine if flagellin administration impacts these antimicrobial activities. Furthermore, the timing of treatment should be investigated to determine if flagellin administration could improve clearance of an established bacterial infection, which would be extremely valuable in the clinical setting.
